# Developing mixed-effects height-diameter model using stand and environmental factors for mixed forests in northern China

**DOI:** 10.1016/j.isci.2025.114446

**Published:** 2025-12-13

**Authors:** Yaxiong Zheng, Yongjie Yue, Runhong Gao, Ram P. Sharma

**Affiliations:** 1 College of Forestry, Inner Mongolia Agricultural University, Hohhot, China; 2 National Field Scientific Observation and Research Station of Greater Khingan Forest Ecosystem, Genhe, China; 3 Institute of Forestry, Tribhuvan University, Kathmandu, Nepal

**Keywords:** environmental science, environmental monitoring, applied sciences

## Abstract

Understanding tree height (H)-diameter at breast height (D) allometric relationship is crucial for estimating biomass, carbon storage, and productivity. Environmental factors and species diversity strongly influence this relationship, yet remain understudied. Using data from 99 plots in Hulunbuir mixed forests, inner Mongolia, we developed an environment-sensitive nonlinear mixed-effects model for five tree species. The model incorporates variables representing stand-level attributes, soil properties, climate factors, and species diversity. Variations at the sample plot and species levels were accounted for by introducing random components into the H-D model. Quadratic mean diameter, total basal area greater than the target D (BAL), Simpson’s diversity index (SIM), mean annual precipitation (MAP), and soil organic carbon significantly affected the H-D relationship. Height growth increased with BAL and MAP but declined with higher SIM. Species traits and environmental factors jointly shaped H-D scaling. The proposed model supports adaptive forest management under changing environments.

## Highlights

• Mixed-effects H-DBH model developed for mixed forests with environmental variables
• Genetic traits and environment jointly shape H-DBH scaling, below theoretical 2/3
• Competition (BAL) and site quality (SOC) increase height; rain and diversity reduce it
• Findings improve biomass and carbon estimates and guide sustainable forest management

## Introduction

Tree height (H) and diameter at breast height (D) are critical metrics frequently used to assess forest growth and development.^1^ These metrics serve important physiological functions: H influences light interception and seed dispersal distance,^2^ whereas D affects water transport and biomass allocation within trees.^3^ Both H and D serve as key predictors for estimating stem volume and forest biomass.^4^^,^^5^ Thus, understanding their functional relationship is essential for quantifying forest productivity.^6^

The allometric relationship between H and D can be described using mathematical functions, typically with two or three parameters.^7^^,^^8^^,^^9^^,^^10^ Functions such as the Mitscherlich, Gompertz, modified Gaussian, log-logistic, and Richards equations are among the most commonly used for modeling the H-D relationship.^7^^,^^9^^,^^11^ These are often referred to as basic H-D models^12^ and can be extended by incorporating additional factors as covariates or predictors. Previous H-D models have primarily focused on single-species natural or plantation forests,^4^^,^^13^^,^^14^ often overlooking the effects of environmental factors such as climate and soil. However, due to the complex species composition and diverse H-D relationships in mixed forests, developing suitable H-D models for these ecosystems is more challenging. For instance, Lei et al.^4^ developed a climate-sensitive stand growth model for *Larix olgensis* plantations in the Changbai mountains, identifying the mean temperature of the driest quarter and precipitation of the wettest month as the most influential climatic predictors for stand basal area and dominant height growth, respectively. Qiu et al.^15^ analyzed the H-D allometry of *Pinus ponderosa* across climate, competition, and diversity gradients in the western United States, finding that tree height was positively correlated with dominant height, mean annual precipitation, and mean annual temperature, but negatively correlated with species mixing. Cudjoe et al.^16^ compared allometric growth and biomass between mixed and monospecific stands, indicating that environmental factors and species differences are key drivers of these patterns.

Understanding the influence of environmental factors on the H-D relationship enables forest managers to develop effective management strategies. Several environmental variables, including temperature, precipitation, wind speed, and soil properties,^17^^,^^18^ have been incorporated into single-species H-D models, thereby enhancing their predictive accuracy. Although these models provide reasonable fits for the sample plots from which data were collected, they are location-specific and may yield substantial errors when applied to tree height predictions in other regions. Variations in the H-D relationship across regions, plots, and species may stem from factors such as stand density, competition, site quality, topography, and climate.^5^^,^^10^^,^^13^ Moreover, the variables selected for modeling vary across regions and species. For example, Jha et al.^18^ using data from the US Forest Inventory and Analysis program identified the stand density index as a key factor influencing the H-D relationship. Other studies have identified wind speed and soil properties as important factors affecting the H-D relationship.

In the early 20th century, commercial overharvesting in the Greater Khingan mountains caused a scarcity of large-diameter trees and a substantial depletion of vegetation resources, leading to the dominance of secondary forests in most stands. Due to the complex forest structure and substantial variation in species diversity within the region, studying H-D relationships in mixed forests has become increasingly challenging. Species diversity reflects the intricate interactions between organisms and their environment, as well as the richness of biological resources in mixed forests.^19^^,^^20^^,^^21^ Although several studies have addressed H-D modeling for mixed forests,^5^^,^^14^^,^^22^ recent research has shown that tree allometry and biomass dynamics differ substantially between mixed and monospecific stands, with species-specific responses to competition and environmental conditions.^16^ Given the uncertainties in our understanding of H-D relationships across regions and species, there is currently a lack of models to predict these relationships in mixed forests based on combinations of stand characteristics, species diversity, and environmental factors.

The Daxing’an mountains in northern China represent a vast and critical ecological barrier. The *Larix gmelinii* forests are primarily distributed in this region, constituting a key component of China’s cold-temperate northern coniferous forests and contributing to the global boreal forest ecosystem. However, since the late 19th century, these forests have experienced significant anthropogenic disturbances, resulting in reduced recoverable resources and severe structural degradation. The quality of Larix forests has declined, with their ecological functions substantially compromised. The destruction and degradation of forest vegetation in the upper Nenjiang River basin, along with extensive structural damage, are major contributing factors.^10^^,^^23^ Therefore, developing H-D models that incorporate stand characteristics and environmental factors is a critical priority for sustainable forest management in the Daxing’an Mountains. Addressing this need is increasingly urgent for China’s forestry sector, as these models can substantially improve the accuracy of forest growth predictions and facilitate informed decision-making in conservation and resource utilization.

This study aims to (1) develop a nonlinear mixed-effects H-D model for mixed forests, incorporating tree- and stand-level characteristics, soil, climate, competition, and species diversity; (2) simulate tree height variations across species along biotic and abiotic gradients; and (3) quantify the impacts of key factors on tree H variations among species. The proposed H-D model will reduce the time and costs associated with sample plot surveys, offer valuable insights for estimating biomass and carbon stocks in mixed forests, and support decision-making in forest management amid environmental changes.

## Results

### Variables selection

**Figure 1:**
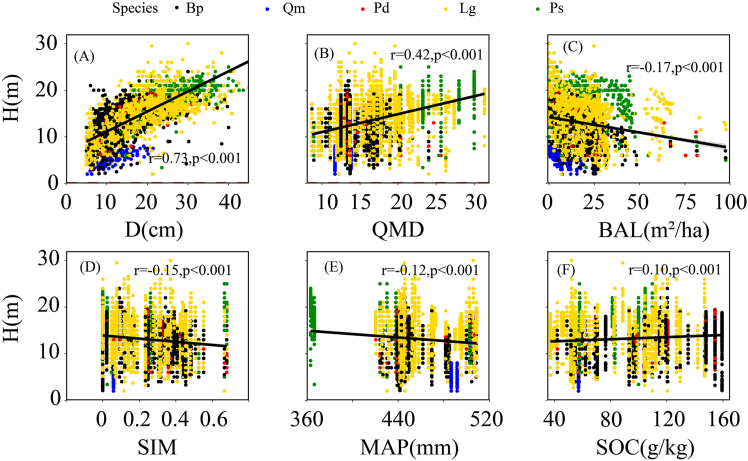
Height plotted against each of the predictor variables retained in the final model for five different tree species Notes: A, diameter at breast height (D); B, quadratic mean diameter (QMD); C, total basal area greater than the target D (BAL); D, Simpson index (SIM); E, mean annual precipitation (MAP); F, soil organic carbon (SOC). *p* value <0.05 indicates a significant correlation.

Using a two-step approach, variables with strong correlations to tree height and without multicollinearity among each other were selected, resulting in six predictors: D, soil organic carbon (SOC), mean annual precipitation (MAP), Simpson’s diversity index (SIM), total basal area greater than the target D (BAL), and quadratic mean diameter (QMD). Figure 1 presents scatterplots of these predictor variables against tree height for each species.

### H-D relationship variation by tree species

**Figure 2:**
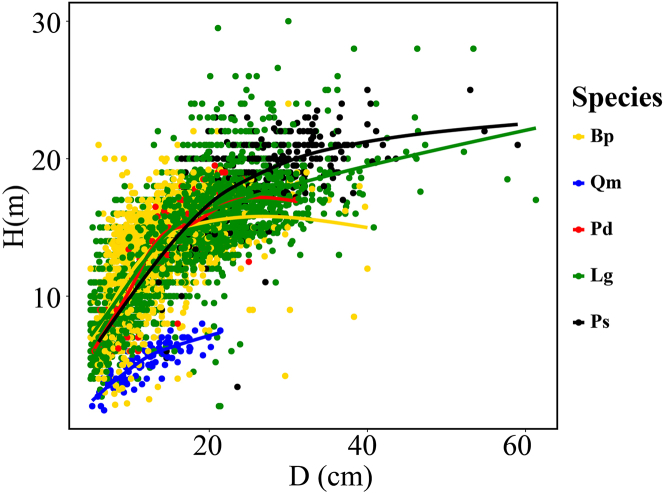
Smoothing curves of tree height overlaid on the scattered plots of the H-D relationship by tree species

**Figure 3:**
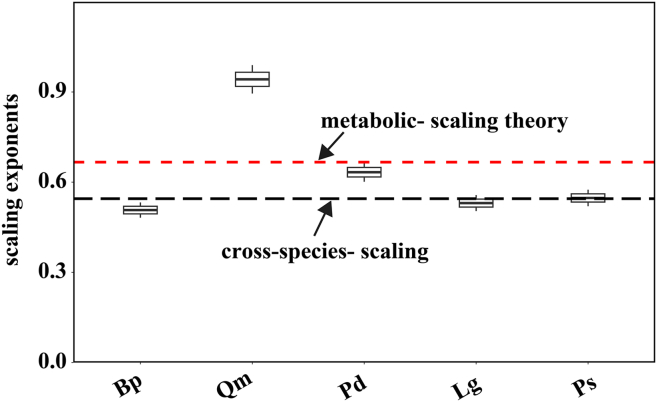
Allometric scaling exponents for different species using Equation 1 Black long-dash line is the cross-species scaling exponent; red two-dash line, metabolic-scaling theory (*H*∝*D*^2/3^).

The scatterplots for five tree species are presented in Figure 2. Different species exhibited varying degrees of fluctuations and differences in the trends, slopes, and variations of H-D. For all species, H-D relationship initially shows a rapid increase followed by a slow increase, resembling the curve fitted by the selected basic model (Equation 1). We plotted the allometric scaling exponents for each species (Figure 3) and this shows each exponent center around to two-third, as predicted by metabolic scaling theory. The scaling exponent for Qm exceeds the theoretical value, while those for other species appear lower, indicating that the allometric relationship between H and D in mixed forests is flexible, and requires an inclusion of additional environmental factors for more accurate description.

### Determinants of H-D variations by species

Based on the principle of minimizing Akaike’s information criterion (AIC), we evaluated combinations of multiple predictor variables and ultimately selected D as the sole individual tree variable. SIM was chosen as a diversity indicator, BAL as a competitive indicator, QMD represents stand developmental stage, MAP as a climate factor, and SOC as a soil factor, with no significant multicollinearity observed. Final model form is as follows:

(Equation 9) $$H_{ijk}=1.3+\beta_{0}D_{ijk}^{\left(\beta_{1}+\beta_{2}SOC_{ij}+\beta_{3}MAP_{ij}+\beta_{4}{\text{SIM}}_{ij}+\beta_{5}BAL_{ijk}+\beta_{6}{\text{QMD}}_{ij}\right)}+\xi_{ijk}$$

where i represents sample plot, j represents tree species, and k represents tree. The predictor variables involved are diameter at breast height (D), soil organic carbon (SOC), mean annual precipitation (MAP), Simpson index (SIM), sum of all basal areas greater than the target D (BAL), and quadratic mean diameter at breast height (QMD). *β*_0_–*β*_6_ are parameters to be estimated.

**Figure 4:**
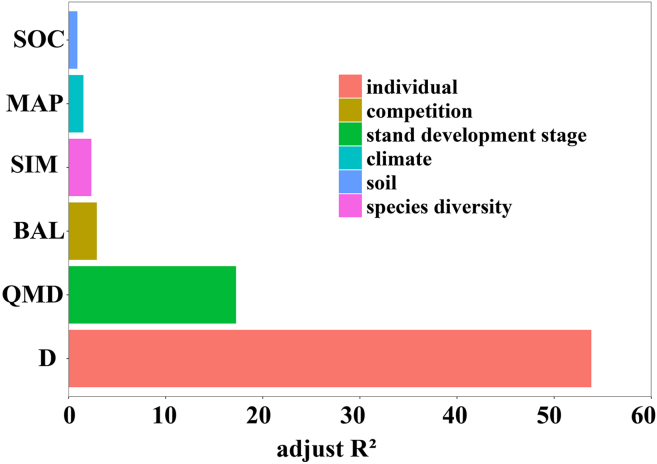
Partial-R^2^ of the H-D model (Equation 9) Definitions of acronyms are the same as in Table 3.

The analysis of explanatory power of the different predictor variables on tree height variations by species revealed following ranking from highest to lowest: D, QMD, BAL, SIM, MAP, and SOC (Figure 4). The H-D relationships across the tree species were influenced not only by development (QMD) but also by the combined effects of soil, competition, climate, and species diversity variables (Figure 4).

### Cross-species nonlinear mixed-effects H-D model

Based on Equation 9, we incorporated random effects both at the sample plot- and species-level to construct non-linerar mixed effect (NLME) H-D model. We considered all possible combinations of fixed and random parameters that held both biological and statistical significance. The models which did not converge or where parameters were not significant (*p* < 0.05) are not presented here (Table S2). Our results show that when the sample level random effects are added to β_0_ and β_1_, and the species level random effects are added to β_6_, the model AIC is the smallest and R^2^ is the largest. The optimal cross-species H-D model effectively explained tree height variations across sample plots and tree species. The form of the NLME H model is as follows:

(Equation 10) $$H_{ijk}=1.3+\left(\beta_{0}+\mu_{i1}\right)D_{ijk}^{\left[\beta_{1}+\mu_{i2}+\beta_{2}{SOC}_{ij}+\beta_{3}{MAP}_{ij}+\beta_{4}{\text{SIM}}_{ij}+\beta_{5}{BAL}_{ijk}+\left(\beta_{6}+\mu_{ij1}\right){\text{QMD}}_{ij}\right]}+\xi_{ijk}$$

where *μ*_*i*1_,*μ*_*i*2_ represents sample plot-level random effects and *μ*_*ij*1_ represents species-level random effects. The remaining parameters and variables are defined as in Equation 9.

We compared the statistical indicators of the models with different parameter combinations. All the parameter estimates are significant (*p* < 0.05), and the scaling exponent β_1_ of the model increased with the inclusion of additional predictor variables and random effects. The incorporation of predictive variables and random effects substantially enhanced the model’s fitting accuracy (Table 1).

**Table 1:** Parameter estimates and statistical indicators for a basic model, OLS model, and NLME model

		Basic model (Equation 1)	OLS model (Equation 9)	NLME model (Equation 10)
Fixed effects	β_0_	2.817 (0.044)	2.092 (0.046)	1.784 (0.092)
	β_1_	0.545 (0.006)	0.802 (0.018)	0.823 (0.237)
	β_2_	–	6.07e-04 (2.55e-05)	-1e-03 (4e-04)
	β_3_	–	−3.14e-04 (2.57e-05)	2e-04 (5e-04)
	β_4_	–	−2.80-e−02 (4e-03)	1.044e-01 (0.073)
	β_5_	–	1e-03 (9.53e-05)	2e-03 (1e-04)
	β_6_	–	-4e-03 (2.71e-04)	-5e-03 (0.003)
Random effects	*μ*_*i*1_	–	–	0.736
	*μ*_*i*2_	–	–	0.110
	*μ*_*ij*1_	–	–	1.6e-03
Statistical indicators	AIC	37,267	36,143	31,827
	R^2^	0.577	0.634	0.818
	RMSE	2.65	2.47	1.73
	TRE	3.81	3.28	1.58

**Notes:** Values in the parenthesis are standard errors, *μ*_*i*1_,*μ*_*i*2_ represents sample plot-level random effects and *μ*_*ij*1_ represents species-level random effects. All other parameters and acronyms are the same as defined earlier. 0.736 represents the random effect of the plot added to parameter β_0_, 0.110 represents the random effect of the plot added to parameter β_6_, and 1.6 × 10^−3^ represents the random effect of the species added to parameter β_2_. These three values correspond to the average random effects across all plots or species.

**Figure 5:**
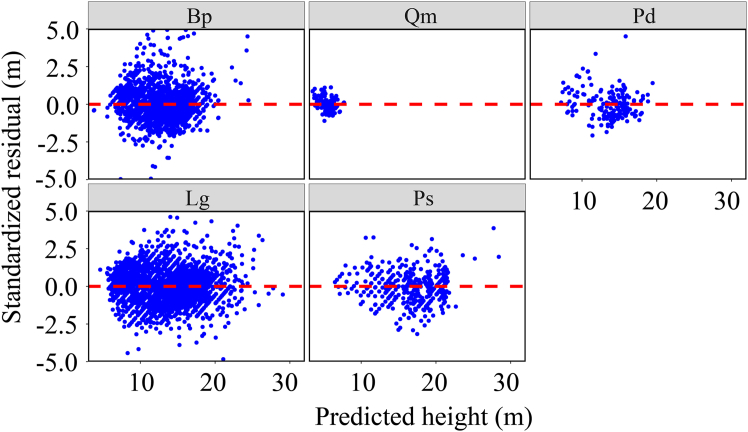
The standardized residuals of Equation 11 for different species

Among the three variance functions evaluated (Equations 3, 4, and 5), the constant plus power function (Equation. 4) most effectively reduced heteroscedasticity (Table 2), resulting in a trend-less residuals (Figure 5). Final NLME model form is as follows:

(Equation 11) $$\left\{ \begin{array}{l} H_{ijk}=1.3+\left(\beta_{0}+\mu_{i1}\right)D_{ijk}^{\left[\beta_{1}+\mu_{i2}+\beta_{2}{SOC}_{ij}+\beta_{3}{MAP}_{ij}+\beta_{4}{\text{SIM}}_{ij}+\beta_{5}{BAL}_{ijk}+\left(\beta_{6}+\mu_{ij1}\right){\text{QMD}}_{ij}\right]}+\xi_{ijk}\\ \xi_{ijk}∼N\left(0,R_{i}=\sigma^{2}G_{i}^{0.5}\Gamma_{i}G_{i}^{0.5}\right)\\ G_{i}=diag\left(\sigma^{2}{\left(\gamma_{1}+D_{ij1}^{{2\gamma }_{2}}\right)}^{2},\ldots ,\sigma^{2}{\left(\gamma_{1}+D_{ijn}^{{2\gamma }_{2}}\right)}^{2}\right) \end{array}\right.$$

**Table 2:** Results of three variance functions (exponential function [Equation 3], constant plus power function [Equation 4], and power function [Equation 5]) of NLME H model

	AIC	BIC	LL	L-ratio	*p*-value
Not added	31,827	31,984	−15,943	–	–
Equation 3	31,814	31,978	−15,935	14.83	<0.0001
Equation 4	31,788	31,951	−15,917	50.82	<0.0001
Equation 5	31,823	31,987	−15,940	5.97	0.014

**Note:** AIC, Akaike’s information criterion; LL, log-likelihood; LRT, likelihood ratio test; BIC, Bayesian information criterion.

### H-D relationship of different species under environmental changes

**Figure 6:**
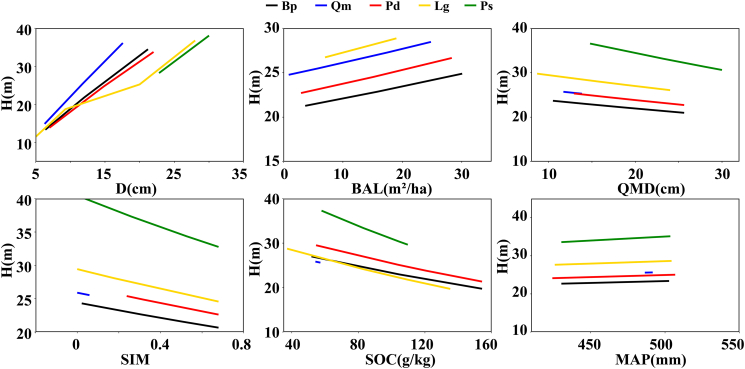
Variations of tree height along the gradients of biotic and abiotic factors for each tree species Each variable is defined in Table 3. To account for the effects of specific predictors, other predictors were set to mean values during the visualization process. The nodes used were the 5th, 50th, and 95th percentiles.

We employed the cross-species NLME H model (Equation 11) to simulate the effects of predictor variables on tree height variations by species, as this approach helps assessing biological reliability of the developed model. The influence of environmental factors aligns with the parameter estimates of the model (Tables 1 and S3). Tree height increased with rise of D, ΒAL, and MAP, while higher values of QMD, SOC, and SIM inhibit tree height variations (Figure 6). Different species exhibit varying responses to the gradient changes of stand and environmental factors.

The slopes of H-D model and environmental factors by species indicate that SIM has the most significant effect on the height of Lg and Βp. The species has the most effect on QMD, while SOC has the smallest effect on the height across the species (Table S3).

## Discussion

**Figure 7:**
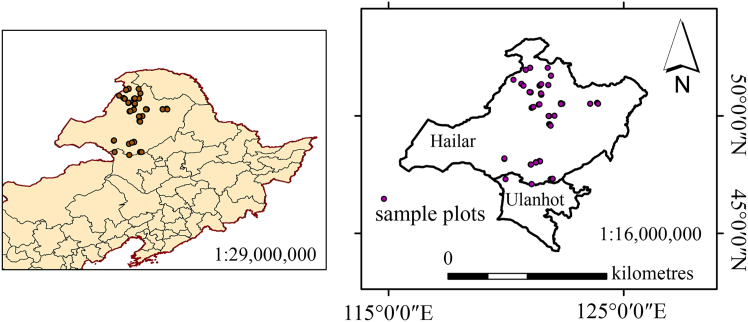
Sample plot locations in the mixed forests

**Figure 8:**
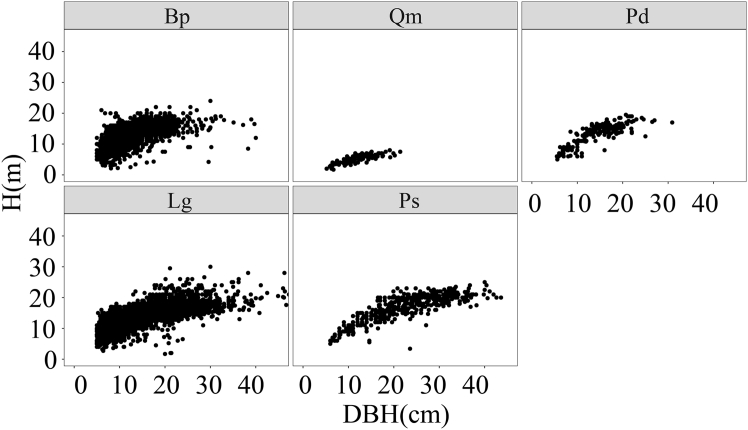
Height plotted against D for different tree species *Betula platyphylla* Sukaczev (Bp); *Quercus mongolica* Fisch. ex Ledeb. (Qm); *Populus davidiana* (Pd); *Larix gmelinii* (Rupr.) *Kuzen* (Lg); *Pinus sylvestris L*. var. *mongolica* Litv. (Ps).

Since the 1970s, rapid economic development coupled with ecological degradation has elevated environmental issues to a primary strategic priority for global society.^24^^,^^25^ As an integral component of terrestrial ecosystems, forest growth is strongly influenced by environmental factors and serves as a sensitive indicator of changes in atmospheric, hydrological, and soil conditions.^26^^,^^27^ Nevertheless, research on the H-D allometric relationship in mixed forests remains limited.^5^^,^^10^^,^^28^ Currently, no studies have investigated the combined effects of climate, soil, and species diversity on the H-D allometric relationship in mixed forests. Insights from such investigations would enhance our understanding of the competitive and facilitative mechanisms governing species growth under evolving environmental conditions and offer a basis for precise biomass and carbon stock assessments in mixed forests. This study established an extensive network of sample plots in temperate coniferous-broadleaf mixed forests across Hulunbuir, Inner Mongolia (Figure 7), characterized by substantial species composition diversity (Tables 3 and 4; Figure 8).

**Table 3:** Statistical indicators of D and tree height for different species

Species	D (cm)				H (m)				numbers
	Min	Max	Mean	STD	Min	Max	Mean	Std	
Bp	5.0	40.0	12.8	4.9	2.1	24.0	12.6	3.5	2,222
Qm	5.2	21.4	11.9	3.6	1.7	8.0	5.2	1.4	166
Pd	5.5	30.9	14.8	4.9	5.0	19.5	13.5	3.4	179
Lg	5.0	61.3	15.2	6.9	2.0	30	13.4	4.1	4,778
Ps	5.0	59.0	22.9	8.7	3.4	25.0	16.8	4.2	440

Note: see Figure 8 for tree species abbreviations.

**Table 4:** Summary statistics for variables of different species, site, soil, and climate

Type	Variables	Min	Max	Mean	Std
Stand variables	DH (m)	7.0	25.7	18.2	3.4
	N (stem/ha)	290	2,667	1,197	486
	BA (m^2^/ha)	0.01	4.36	0.26	0.27
	BAL (m^2^/ha)	0.00	97.78	15.13	10.68
	MD (cm)	8.3	31.2	14.9	4.4
	QMD (cm)	8.7	31.4	15.7	4.4
	CD	0.40	0.90	0.70	0.11
Soil variables	BD (g/cm^3^)	0.59	1.20	0.91	0.12
	TN (g/kg)	2.69	14.40	9.35	3.14
	SOC (g/kg)	37.20	159.30	90.16	34.47
	pH	5.5	7.0	6.11	0.30
	Sand (%)	28.00	50.00	40.16	4.07
	Silt (%)	29.00	45.00	38.79	3.86
	Clay (%)	2.70	4.20	3.39	0.26
Site condition variables	Elevation (m)	423.80	1,135	791.10	167.88
	SL	0	47	11	9
	SIE	−6.71	6.90	−0.21	4.35
	CIE	−6.77	6.73	−0.21	5.02
Climate variables	MAT	−4.57	−0.17	−2.94	1.03
	MWMT	16.98	19.56	18.24	0.70
	MCMT	−28.90	−21.89	−26.77	1.68
	TD	41.03	46.42	45.01	1.31
	MAP	363.40	508.80	454.10	27.55
	AHM	12.44	24.18	16.33	2.24
	DD_0	2,380	3,419	3,070	240
	DD5	1,066	1,515	1,262	120
	DD_18	6,645	8,149	7,591	348
	DD18	30.11	103.31	58.65	19.85
	NFFD	108.3	140.1	123.4	8.2
	PAS	36.98	74.71	58.85	8.50
	EMT	−45.15	−36.38	−42.10	2.16
	EXT	31.31	33.03	32.09	0.42
	Eref	536.0	658.4	575.0	28.2
	CMD	162.7	293.1	205.6	21.4
	M	43.51	84.33	65.36	8.30
Diversity index	SIM	0.00	0.68	0.21	0.19
	SHI	0.00	9.78	2.83	2.29

**Notes:** All acronym are the same as defined in Table 3; M = MAP/(MAT +10). SIE = sin (SL) × ln (EL), CIE = cos (SL) × ln (EL). When the plot is a pure forest, the species diversity index is 0; when there is no vegetation under the forest, the vegetation diversity is recorded as 0.

To precisely characterize the influence of environmental factors on the H-D allometric relationship in mixed forests, five categories of variables were incorporated. The base model (Equation 1) was expanded by incorporating covariates. Stand-level variables, including BAL and QMD, were employed to quantify interspecific competition and stand developmental stage.^5^ SOC serves as a robust indicator of soil fertility in the study area.^29^^,^^30^ MAP is a widely utilized climatic variable that captures precipitation variability within sample plots.^10^^,^^15^^,^^31^ Furthermore, diversity indices were incorporated to enhance predictions of the H-D allometric relationship in mixed forests (Table 5; Figure 1).

The incorporation of QMD and BAL among the selected variables enhanced the model’s fitting accuracy (Table 5). Prior studies have demonstrated that incorporating BAL improves the accuracy of H-D models.^10^^,^^32^ QMD comprehensively captures stand diameter structure and mean competition intensity, functioning as a pivotal indicator of stand developmental stage and structural stability.^33^^,^^34^^,^^35^ Variations in QMD exert a substantial regulatory influence on tree height growth in mixed forests. At low to moderate QMD levels, stand structure is optimized, competition is balanced, and species exhibit vertical stratification, thereby enhancing light-use efficiency and accelerating height growth. As QMD increases, dominant trees occupy the upper canopy, whereas understory individuals experience light limitation, thereby intensifying competition and decelerating height growth.^36^ Thus, QMD demonstrates a nonlinear, bidirectional effect on tree height growth in mixed forests, where moderate competition facilitates growth, while excessive competition inhibits it. Consequently, QMD serves as a crucial ecological indicator connecting stand structure to functional processes.

**Table 5:** Statistical indicators for different variables added to the basic model (Equation 1)

Model	Variables	Added variable location		AIC	LL	R^2^
		β_0_	β_1_			
Basic	D	–	–	37,267	−18,630	0.577
M1	basic+ developmental stage	–	D + QMD	37,178	−18,585	0.582
M2	M1+ competition	–	D + QMD + BAL	37,004	−18,497	0.591
M3	M2+ species diversity	–	D + QMD + BAL + SIM	36,927	−18,458	0.595
M4	M3+ climate	–	D + QMD + BAL + SIM + MAP	36,685	−18,335	0.608
M5	M4+ soil	–	D +QMD + BAL + SIM + MAP + SOC	36,143	−18,064	0.634

**Notes:** Diameter at breast height (D), soil organic carbon (SOC), mean annual precipitation (MAP), Simpson index (SIM), the sum of all basal areas greater than the target D (BAL), and quadratic mean diameter at breast height (QMD), Akaike’s information criterion (AIC), log-likelihood (LL).

This study also evaluated the impact of species diversity on the H-D allometric relationship. SIM emerged as the most effective metric for representing species diversity, capturing both species richness and relative abundance within sample plots.^37^^,^^38^^,^^39^ SIMSP exhibited inhibitory effects on tree height (Figure 6; Table S3). Cudjoe et al.^16^ provide direct empirical evidence, documenting contrasting allometric responses between pine and oak in mixed versus monospecific stands. This pattern can be attributed to heightened interspecific competition as diversity increases, wherein contests for light, water, and nutrients limit the vertical growth potential of individual trees.^40^ Additionally, interspecific differences in shade tolerance and growth strategies may lead to greater resource allocation toward radial growth or root development for enhanced physiological stability, rather than sustained height increment.^41^ Furthermore, increased canopy structural complexity may aggravate uneven light distribution, thereby diminishing light-use efficiency in dominant trees. Overall, although elevated species diversity fosters resource complementarity and stand stability, it may concurrently lead to reduced mean tree height owing to trade-offs in energy allocation and growth strategies.^36^ Similar patterns have been documented in temperate mixed forests, where Scots pine and sessile oak exhibit contrasting H-D and biomass responses in mixed versus monospecific stands.^16^^,^^34^

In certain studies, incorporating dominant height has been shown to substantially enhance model accuracy.^10^ Presently, no standardized definition or calculation method exists for estimating dominant height in mixed forests.^14^ We employed the widely used soil variable, SOC, to represent site conditions (Table 5; Figure 4). SOC accumulation is modulated by climatic factors such as temperature and precipitation; elevated temperatures accelerate decomposition, while precipitation affects organic matter inputs and soil moisture. In turn, SOC variations influence nutrient availability and water retention, thereby modulating tree growth patterns in mixed forests and shaping the H-D allometric relationship.^42^

For each sample plot, climate data were aligned with stand age by ascertaining the ages of dominant trees. The model was subsequently extended using these corresponding climate data.^4^^,^^15^ Employing mean climate data simplifies the model and minimizes data demands; however, it overlooks seasonal variations, extreme events, interannual variability, and local microclimatic differences, potentially introducing biases in tree growth predictions. Therefore, future research should integrate growing-season climate data, extreme event indices, or annual climate records to enhance model accuracy and applicability. Species-specific differences also result in variations in tree height trajectories under comparable climatic condition (Figure 6; Table S3). Increases in precipitation generally promote height growth across tree species. Under adequate water availability, trees typically attain greater heights.^43^

When the cross-species scaling exponent in the H-D allometric model falls below the theoretical value of two-third (Figure 3), it underscores the modulating effects of environmental and biological factors on allometric relationships. This deviation arises from a confluence of factors, including resource limitations (e.g., water and nutrients), light availability and stand density, temperature and elevation, as well as biological traits. Although metabolic scaling theory predicts higher height-diameter exponents in fast-growing species due to preferential height investment,^44^^,^^45^^,^^46^^,^^47^ our results showed that the slow-growing *Quercus mongolica* had the largest exponent (≈0.91). This likely reflects species-specific strategies rather than a deviation from theory. Slow-growing species with dense wood and high mechanical stability can maintain tall, slender forms at small diameters. In competitive, shaded environments, *Qm* allocates more biomass to height for light capture, producing a steeper height-diameter relationship.^41^ Thus, the higher exponent of oak indicates an adaptive response to light competition and structural trade-offs, suggesting that scaling variation among species depends on ecological strategy and environmental conditions rather than intrinsic growth rate.

### Limitations of the study

Any H-D model may encounter limitations, particularly when applied to novel or unanticipated forest conditions, potentially yielding inaccurate estimates of the target variable.^48^^,^^49^ This study focused exclusively on mixed forests in Inner Mongolia’s primary forested regions; consequently, the results primarily reflect the ecological characteristics of this area, and caution is advised when extrapolating to larger scales or different forest types. The developed H-D model exhibited robust predictive performance (Equation 11). Nevertheless, recalibration is essential prior to application to ensure accuracy, especially given that larger datasets often introduce increased variability. Future research should gather multi-source data from wider geographical areas and incorporate long-term observational and experimental datasets to validate and refine the model further.

## Resource availability

### Lead contact

Requests for further information and resources should be directed to and will be fulfilled by the lead contact, Yongjie Yue (wolongyue@126.com).

### Materials availability

This study did not generate new materials.

### Data and code availability

All data generated or analyzed during this study are included in the manuscript and supplementary tables.

This article does not report original code.

Any additional information required to reanalyze the data reported in this article is available from the lead contact upon request.

## Acknowledgments

This research was funded by the 10.13039/501100004763Inner Mongolia Natural Science Foundation (2025QN03020), the 10.13039/501100011811Inner Mongolia Agricultural University High-Level/Outstanding PhD Talent Introduction Research Startup Project (NDYB2022-14), and the Inner Mongolia Autonomous Region 2022 Autonomous Region-Level Public Institutions High-Level Talent Introduction Research Support Fund (DC2300001273).

## Author contributions

Investigation, Y.Y. and R.G.; data curation, Y.Y. and R.G.; software, Y.Z. and R.P.S.; writing – original draft preparation, Y.Z.; writing – review and editing, Y.Z., R.P.S., and R.G.; funding acquisition, Y.Z.

## Declaration of interests

The authors declare no competing interests.

## STAR★Methods

### Key resources table

**Table:** 

REAGENT or RESOURCE	SOURCE	IDENTIFIER
**Deposited data**		

Climate Data	This paper	http://climateap.net

**Software and algorithms**		

R Project 4.4.1	R Foundation	https://www.r-project.org/

### Method details

#### Study area

Forest surveys and measurements were conducted in the Daxing’an Mountains (47°5'10"–52°2'48" N, 119°55'42"–123°54'55" E), located in north-eastern China (Figure 7). This region, situated in Inner Mongolia, features gently undulating terrain, with elevations decreasing from west to east. The western portion is characterized by cold, permafrost-dominated landforms typical of high-latitude regions, whereas the eastern portion experiences a cold, humid monsoon climate in the cold-temperate zone. Winters are long and cold, lasting over nine months, while summers are short and warm, lasting less than one month. The frost-free period ranges from 70 to 100 days. Average annual precipitation is 442 mm, with 85–90% occurring during the summer months (June–August) and 10% falling between late October and early April, typically as snow. Average annual relative humidity is 70%. The annual temperature range is substantial, with an average annual temperature between –2°C and –4°C. January temperatures average –20°C to –30°C, while July temperatures range from 17°C to 20°C. The region is predominantly cold-temperate coniferous forest, with *Larix gmelinii* (Rupr.) *Kuzen* (Lg) as the dominant tree species. Other associated species include *Betula platyphylla* Sukaczev (Bp), *Pinus sylvestris L*. var. *mongolica* Litv. (Ps), *Populus davidiana* (Pd), and several shrubs, such as *Rhododendron dauricum* and *Rhododendron tomentosum*. These forests are representative of the region and exhibit distinctive regional characteristics.

#### Sampling and measurements

To comprehensively assess the influence of climate on the H-D allometric relationship in northern temperate coniferous and broad-leaved mixed forests, the sampling design was based primarily on the major forest regions of Hulunbuir. In accordance with principles of representativeness and randomness, 99 sample plots were established in mixed coniferous–broadleaf forests in Genhe, Yakeshi, Arxan, Ulanhot, and Alihe in Hulunbuir. All sample plots were rectangular, with areas of primarily 0.06, 0.1, or 0.25 ha. Among them, 33 plots were remeasured based on previously established plots (each 30 × 20 m). In areas with complex terrain or uneven vegetation, plot sizes were expanded as necessary to better capture forest stand structure and species diversity (Figure 7). In each plot, trees with D ≥ 5 cm were recorded, including their D, height, and crown closure; additionally, the number of tree species with D < 5 cm was noted to facilitate subsequent calculation of species diversity. Canopy density data were obtained using a fisheye lens, while latitude, longitude, and elevation were measured with a GPS device. Slope was determined using a compass. The tree species were identified using the Plant Science Center database (https://www.Plantplus.cn/cn). Ultimately, five major tree species, comprising 7,786 trees, were selected for H-D modeling. Species with insufficient data were excluded from the analysis. Figure 8 presents scatterplots of H against D by species. Pearson correlation regression analysis was conducted on the variables influencing the HCB. And the least significant difference (LSD) test was applied to separate means, with *p* < 0.05 considered statistically significant (Figure 1).

Table S1 lists species names and counts within the sample plots. To account for the impact of tree species diversity, commonly used indices (Shannon and Simpson) were employed to quantify species richness and evenness.^50^^,^^51^

#### Climate data

Based on latitude, longitude, and elevation of each plot, 16 annual climate variables were obtained using ClimateAP software (See http://climateap.net, December 2024).^52^ Increment cores from dominant trees in each plot were extracted using an increment borer to determine tree ages. Historical climate variables for the past 100 years (1918–2017) were retrieved for each plot, and dominant tree ages were matched with corresponding meteorological data. Mean values of these matched data were used to represent climatic conditions in the study area. For each plot, climatic variables were averaged over the stand's dominant height age. For example, if a stand's dominant height age was 35 years, mean climate data over those 35 years were used for that plot. This approach ensures that climatic conditions correspond to the period of primary stand growth, thereby enhancing the ecological relevance of environmental covariates in the H-D models. The de Martonne aridity index (M = MAP / (MAT + 10)) was used as a measure of humidity conditions in the sample plots. These climate variables provide a straightforward description of plot meteorological characteristics and are widely used in model development.^10^^,^^31^^,^^53^ Definitions of stand, soil, and climate variables are provided in Table 3; descriptive statistics are summarized in Table 4; and Table 3 presents statistics for diameter at breast height (D) and tree height by species.

**Table 3:** Definitions of variables describing stands, soil, site, and climate

Variables	Definition
D (cm)	Diameter at breast height
H (m)	Height
DH (m)	Dominant height
BA (m^2^/ha)	Basal area
BAL (m^2^/ha)	The sum of all basal area greater than the target D
MD (cm)	Mean diameter at breast height
QMD (cm)	Quadratic mean DBH
CD	Canopy density
BD (g/cm^3^)	Bulk density
TN (g/kg)	Total nitrogen concentration
SOC (g/kg)	Soil organic carbon concentration
pH	Pondus Hydrogenii
Sand (%)	Diameters between 0.05 for fine sand to 2.0 mm
Silt (%)	A medium size particle falling between 0.002 and 0.05 mm in size
Clay (%)	The smallest being less than 0.002 mm in size
EL (m)	Elevation
SL (°)	Slope
MAT (°C)	Mean annual temperature
MWMT (°C)	Mean warmest month temperature
MCMT (°C)	Mean coldest month temperature
TD (°C)	Temperature difference between MWMT and MCMT, or continentality (°C)
MAP (mm)	Mean annual precipitation
AHM(°C)	Annual heat (MAT+10)/(MAP/1,000)
DD_0 (°C)	Degree-days below 0°C, chilling degree-days
DD5 (°C)	Degree-days above 5°C,growing degree-days
DD_18 (°C)	Degree-days below 18°C, heating degree-days
DD18 (°C)	Degree-days above 18°C, cooling degree-days
NFFD	The number of frost-free days
PAS	Precipitation as snow (mm) between August in previous year and July in current year
EMT	Extreme minimum temperature
EXT	Extreme maximum temperature
Eref	Hargreaves reference evaporation
CMD	Hargreaves climatic moisture deficit
SIM	Simpson index
SHI	Shannon index

#### Soil physicochemical properties

Three soil samples were collected from 0-30 cm depth in each plot using a soil auger between July and September 2017. The samples were mixed to form a composite sample (∼1 kg) and transported to the laboratory for analysis. Samples were air-dried, with roots and debris removed prior to analysis. Nutrient content was determined using a SEDIMAT4-12 Analyzer. Soil samples were sieved through a 2 mm mesh to measure soil organic carbon (SOC), total nitrogen (TN), and total phosphorus (TP). SOC concentration was determined using the potassium permanganate external heating method and the dry combustion method (Multi N/C 2100).^54^ TN was analyzed using the H_2_SO_4_ digestion method and the Kjeldahl nitrogen determination method (FoodALYT D5000).^55^ TP was measured using the H_2_SO_4_–HCLO_4_ digestion method and the molybdenum-antimony colorimetric method (Rayleigh UV-2601).^54^ pH was determined using potentiometry. Definitions of soil variables are provided in Table 3, with summary statistics in Table 4.

#### Model development

The H-D relationship is largely influenced by site and stand characteristics, species diversity, and environmental factors. This relationship may exhibit varying trends and shapes. Numerous studies have examined the impact of model parameters on this relationship.^5^^,^^6^^,^^56^ Although increasing the number of parameters can enhance fitting accuracy, improvements may be minimal or negligible in practice. Furthermore, excessive input variables and overparameterization may cause convergence issues.^10^ Two-parameter models, such as the power function (Equation 1), are widely used due to their simplicity and interpretability, making them a common choice in forest modeling.^10^^,^^13^^,^^15^

(Equation 1) $$H_{ijk}=1.3+\beta_{0}D_{ijk}^{\beta_{1}}+\xi_{ijk}$$

where *H*_*ijk*_ and *D*_*ijk*_ represent height and diameter at breast height of the *k* th tree in the *j* th species, nested within the *i* th sample plot; *β*_0_ is a scaling coefficient, and *β*_1_ is the scaling exponent, and *ξ*_*ijk*_ is the error term.

Our modeling approach is based on metabolic scaling theory, which posits a fixed allometric exponent (approximately 2/3) governing the H-D relationship. This has been validated across numerous tree species.^44^^,^^45^^,^^46^^,^^47^ The scaling exponent reflects proportional relationships among morphological and functional traits, revealing plant adaptive strategies for resource acquisition and energy allocation. The exponent's magnitude indicates trade-offs in response to environmental conditions: higher values suggest rapid-growth strategies with high resource-use efficiency, whereas lower values indicate conservative strategies adapted to stressful environments. Variations in exponents among species or communities also indicate differences in ecosystem structure and functional stability.

#### Selection of factors affecting the H-D relationship

Variable selection considered both fitting accuracy and biological significance. Previous studies have evaluated numerous factors related to tree height as candidate predictors. These can be categorized into five groups: (1) stand variables, (2) site variables, (3) climate variables, (4) soil variables, and (5) species diversity indices.

(1) Stand variables include basal area of larger trees (BAL; competition), quadratic mean diameter (QMD; stand development), mean diameter (MD), crown density (CD), and basal area (BA).
(2) Site variables: To examine tree height responses under varying site conditions, common topographic variables were selected, including elevation (EL, m) and slope (SL, °). The Stage transformation was applied to combine EL and SL, capturing their interaction more effectively than original variables alone.^57^ Specifically, the sine of SL was combined with the natural logarithm of EL to form SIE, defined as SIE = sin (SL) × ln (EL), and the cosine of SL was combined similarly to form CIE, defined as CIE = cos (SL) × ln (EL). Dominant height (DH) was calculated as the arithmetic mean of maximum tree heights per species per plot.^5^
(3) Climate variables: The impact of 17 bioclimatic variables on tree height was evaluated.
(4) Soil variables: Although soil properties can affect height responses to climate change,^58^ only their impact on H was considered here.
(5) Diversity indices: Species diversity was quantified primarily using the Shannon (SHI) and Simpson (SIM) indices, evaluating their potential impact on the H-D relationship.

A two-stage approach^59^ was used to select key predictors, with variance inflation factor (VIF ≤ 5) applied to minimize multicollinearity. Preliminary fitting of tree height data by species was performed using the nlsList function in R. Correlation analysis then examined relationships between model coefficients and potential covariates. Key predictors were selected based on these results and VIF (≤ 5) to avoid multicollinearity.

#### Two-level nonlinear mixed-effects H-D model

A two-level nonlinear mixed-effects (NLME) tree height model was developed using the base model (Equation 1), incorporating plot- and species-level random effects. All possible combinations of fixed parameters and random effects were considered. The optimal form was selected based on Akaike information criterion (AIC) and log-likelihood (LL).^60^ A likelihood ratio test was performed to avoid overparameterization.

To address heteroscedasticity and autocorrelation in the variance-covariance matrix (**R**):

(Equation 2) $$R_{i}=\sigma^{2}G_{i}^{0.5}\Gamma_{i}G_{i}^{0.5}$$

where *σ*^2^ is the scaling factor for error dispersion, corresponding to the residual variance of the estimated model; **G**_*i*_ is an *n*_*i*_×*n*_*i*_ diagonal matrix of the within sample plot heteroscedasticity variances; and Γ_*i*_ is an *n*_*i*_×*n*_*i*_ matrix representing heteroscedasticity variances within each sample plot; and **R** is a matrix that describes the autocorrelation structure of errors within the sample plot. Our preliminary analysis revealed a significant heteroscedasticity issue, which was addressed by one of the three variance functions (Equations 3, 4, and 5) that we evaluated. The best model form was selected based on the AIC.

(Equation 3) $$Var=\sigma^{2}\;\exp\left(2\gamma D_{ijk}\right)$$

(Equation 4) $$Var=\sigma^{2}{\left(\gamma_{1}+D_{ijk}^{2\gamma_{2}}\right)}^{2}$$

(Equation 5) $$Var=\sigma^{2}D_{ijk}^{2\gamma }$$

where *D*_*ijk*_ is the D in the *k* th tree in the *j* th species nested in the *i* th sample plot; and *γ*, *γ*_1_, and *γ*_2_ are parameters to be estimated, and *σ* is the residual variance of the NLME H-D model.

This study focused on effects of variables such as stand characteristics, climate, soil, and species diversity on the H-D relationship in mixed forests; thus, model calibration or random effects estimation for height prediction was not considered. Three common statistical indicators (Equations 6, 7, and 8) were used to evaluate NLME H model prediction accuracy.

(Equation 6) $$R^{2}=1-\sum_{i=1}^{n}{\left(H_{Oijk}-H_{Eijk}\right)}^{2}/\sum_{i=1}^{n}{\left(H_{Oijk}-\frac{\sum_{i=1}^{n}H_{Oijk}}{n}\right)}^{2}$$

(Equation 7) $$RMSE=\sqrt{\frac{1}{n}\sum_{i=1}^{n}{\left(H_{Oijk}-H_{Eijk}\right)}^{2}}$$

(Equation 8) $$TRE=\sum_{i=1}^{n}\left(H_{Oijk}-H_{Eijk}\right)/\sum_{i=1}^{n}H_{Eijk}*100\%$$

where *H*_*Eijk*_ is the estimated tree height in the *k* th tree in the *j* th species nested in the *i* th sample plot, *H*_*Oijk*_ is the observed tree height in the *k* th tree in the *j* th species nested in the *i* th sample plot, n is the number of total observations. RMSE: root mean square error, TRE: total relative error and R^2^: coefficient of determination.

NLME H-D model parameters were estimated using maximum likelihood with the Lindstrom-Bates algorithm, implemented in the nlme package of R (version 3.6.3). The Lindstrom-Bates algorithm and nlme package are described in the literature.^31^^,^^61^^,^^62^

### Quantification and statistical analysis

Quantification and statistical analysis are mentioned in the method details section.
