# Supplementary material for: Developing mixed-effects height-diameter model using stand and environmental factors for mixed forests in northern China
Source: iScience. 2025 Dec 13;29(1):114446. doi: 10.1016/j.isci.2025.114446 (PMC12809735; doi:10.1016/j.isci.2025.114446)
Supplement: Document S1. Tables S1–S3 [file mmc1.pdf]

iScience, Volume *29*

Supplemental information

Developing mixed-effects height-diameter model using stand and environmental factors for mixed forests in northern China

Yaxiong Zheng, Yongjie Yue, Runhong Gao, and Ram P. Sharma

Table S1. Different tree species in English, Latin, and abbreviations

English name	Latin	abbreviations	number
**white birch**	*Betula platyphylla* Sukaczev	Bp	2222
**Mongolian oak**	*Quercus mongolica* Fisch. ex Ledeb.	Qm	166
**aspen**	*Populus davidiana*	Pd	179
**larix gmelinii**	*Larix gmelinii*（Rupr.）Kuzen	Lg	4778
**Mongolian scots pine**	*Pinus sylvestris* L. var. *mongolica Litv.*	Ps	440
**black birch**	*Betula dahurica Pall.*	Bd	10
**red birch**	*Betula albosinensis Burk.*	Ba	2
**spruce**	*Picea asperata Mast.*	Pa	3

Table S2 Combination forms of different random parameters

Sample plot	species	AIC	R^2
β_0	β_0	32236	0.800
β_0	β_1	31946	0.817
β_0	β_6	31960	0.817
β_1	β_0	32018	0.817
β_1	β_1	32540	0.790
β_1	β_6	32534	0.790
β_6	β_0	32036	0.818
β_6	β_1	32564	0.790
β_6	β_6	32560	0.791
β_0,β_1	β_0	31859	0.818
β_0,β_1	β_1	31842	0.818
**β_0,β_1**	**β_6**	**31827**	**0.818**
β_1,β_6	β_0	32020	0.817
β_1,β_6	β_1	32542	0.790
β_1,β_6	β_6	32536	0.790

β_0,β_6	β_0	31876	0.818
β_0,β_6	β_1	31858	0.818
β_0,β_6	β_6	31843	0.818

Table S3 Slope of H-D model by species under different environmental factors

**species**	**Number**	**Slope**				
		SOC	**MAP**	**BAL**	**SIMSP**	**QMD**
**Bp**	2222	-0.0003	0.0006	0.0015	-0.131	-0.0059
**Qm**	166	-0.0001	0.0032	0.0020	-0.021	-0.0017
**Pd**	179	-0.0005	0.0002	0.0010	-0.0292	-0.0012
**Lg**	4778	-0.0005	0.0007	0.0011	-0.043	-0.0056
**Ps**	440	-0.0003	0.0001	0.0017	-0.0342	-0.0001
